# “Deservingness” and Public Support for Universal Public Goods: A Survey Experiment

**DOI:** 10.1093/poq/nfad007

**Published:** 2023-04-03

**Authors:** Thomas Gift, Carlos X Lastra-Anadón

**Affiliations:** Associate Professor, Department of Political Science, University College London, London, UK; Associate Professor, School of Politics, Economics and Global Affairs, IE University, Madrid, Spain

## Abstract

Voters support less spending on means-tested entitlements when they perceive beneficiaries as lacking motivation to work and pay taxes. Yet do concerns about the motivations of “undeserving” beneficiaries also extend to universal public goods (UPGs) that are free and available to all citizens? Lower spending on UPGs poses a particular trade-off: it lessens subsidization of “unmotivated” beneficiaries, but at the expense of reducing the ideal levels of UPGs that voters personally can access. Studies suggest that individuals will sacrifice their preferred amounts of public goods when beneficiaries who do not pay taxes try to access these goods, but it is unclear whether they distinguish based on motivations. To analyze this question, we field a nationally representative survey experiment in the UK that randomly activates some respondents to think about users of the country's universal National Health Service as either “motivated” or “unmotivated” noncontributors. Although effect sizes were modest and spending preferences remained high across the board, results show that respondents support less spending on the NHS when activated to think of users as “unmotivated” noncontributors. These findings suggest how the deservingness heuristic may shape public attitudes toward government spending, regardless of whether benefits are targeted or universal.

Shortly after becoming UK prime minister in 2019, Boris Johnson’s past words came back to haunt him. An article he had penned more than two decades prior was unearthed (Bienkov 2019), in which he observed the “moral point” that “if NHS services continue to be free in this way, they will continue to be abused like any free service” (Johnson 1995). In calling for structural reform of the National Health Service and emphasizing the term “abuse,” the language sparked criticism (Glaze 2019; Topple 2020). As a politician today, Johnson is much more likely to be heard praising the NHS as “sacred” and lauding “the simple beauty of the principle that … if you fall sick the whole country gathers figuratively at your bedside” (Johnson 2019). Yet his earlier comments potentially vocalized a common, if often unspoken, opinion. A strong belief in universal healthcare belies an underlying concern about “undeserving” beneficiaries.

A robust finding in political economy is that voters support less generous welfare states when they perceive users as lacking motivation to work and pay taxes (Gilens 1999; van Oorschot 2000; Appelbaum 2001; Petersen et al. 2011; Petersen 2012; DeSante 2013; van Oorschot and Meuleman 2014). Yet do such concerns affect not only support for means-tested entitlements, but also for universal public goods (UPGs)—such as public healthcare—that are free and available to everyone? Research shows that voters consider both their own self-interest and the characteristics of likely beneficiaries from social policies (Cavaillé and Trump 2015). However, this distinction poses a particular trade-off when assessing UPGs that may have a redistributive component, but which also yield benefits to all voters: lower spending on these goods diminishes benefits to the “unmotivated,” but at the cost of eroding services for everyone.

Studies reveal that citizens will reduce their ideal support for UPGs when “undeserving” beneficiaries who do not pay into the tax base try to access these goods (Yamagishi 1986; Ostrom, Walker, and Gardner 1992; Fehr and Gachter 2000, 2002; Smirnov 2007; Osborne 2009; Rand, Ohtsuki, and Nowak 2009). Yet the focus is typically on their contributions only. As with welfare, however, some “undeserving” beneficiaries may be “motivated” but still not pay into the tax base through no fault of their own (for example, if they are disabled, sick, or got laid off), whereas others may be simply “unmotivated” to exert effort.^1^ It is unclear whether voters will support even lower spending on UPGs for “unmotivated” noncontributors when considering goods that they themselves consume. In this article, we analyze whether voters adjust their support for UPGs when activated to think of the motivations of noncontributors.

From a theoretical standpoint, we develop the concept of the UPG trade-off in public opinion and analyze how citizens react to the motivations of “undeserving” beneficiaries. From a policy standpoint, the question has relevance for the sustainability of UPG expenditures, particularly in advanced democracies. In healthcare, for example, even before COVID-19, where aging and new medical technologies have spiked costs, expenditures were set to grow at 2.7 percent yearly in the period 2020–2030 in the OECD, faster than GDP (OECD 2019). Public support for such increases may be fragile, however, as polarized media or political actors emphasize negative traits of beneficiaries. This is especially salient given claims of mounting anti-solidarity trends in many post-industrialized democracies (Roemer et al. 2007) that threaten social cohesion (Luttmer 2001; Shayo 2009; Lupu and Pontusson 2011).

We conceptualize two main ways by which voters may reduce support for UPGs when activated to think of “undeserving” beneficiaries. First, *contributions-only-based preferences* take into account only the contributions of beneficiaries to the tax base. In line with prior research, voters may support less spending on UPGs when beneficiaries are noncontributors. Voters, however, should not demand either greater or fewer departures from their ideal levels of spending based on the motivations of noncontributors. Conversely, *motivations-based preferences* consider not only the contributions of beneficiaries, but also their motivations. By this logic, voters may again support less spending on UPGs when noncontributors try to access goods. However, voters should support the least spending and be most willing to reduce their preferred levels of support when noncontributors are “unmotivated.”

To adjudicate between these responses, we conduct a nationally representative survey experiment in the UK that presents fictional users of the country’s National Health Service (NHS) and then asks respondents how much spending they prefer on the NHS. We randomly activate some respondents to think about NHS users as noncontributors to the income tax base, varying their levels of motivation. By “motivated,” we depict users looking for work who are capable of functioning in the labor market (e.g., not disabled or sick). By “unmotivated,” we depict users unconcerned with looking for work, but who still feel entitled to NHS services. The presentations—designed to simulate portrayals by politicians, the media, or activists (Zucchino 1999; Katz 2013; Romano 2018)—speak to core precepts of social reciprocity, where motivation is broadly defined as the willingness of recipients to contribute effort and resources in exchange for benefits (Fong, Bowles, and Gintis 2006; Petersen 2015).

The UK is a useful case because notions of “deservingness” have long shaped public conversations over government policy, and data reveal that large percentages of citizens think “unmotivated” beneficiaries receiving state support is a problem (NatCen Social Research 2015). We focus on the NHS because virtually all UK citizens access the NHS, making it a paradigmatic UPG. Furthermore, healthcare is one of the UK’s largest budgetary outlays (amounting to almost 10 percent of GDP in 2018 [The King’s Fund 2019b]), meaning it is a high-order fiscal priority with large political stakes. Finally, broad cross-partisan support exists for increasing (or at least maintaining) commitment to a strong NHS (Wellings 2017). To the degree that changing the motivations of users shifts public support for the NHS, we might expect such effects to be at least as significant when applied to other UPGs where support is less solidified.

We find evidence of *motivation-based preferences*. Respondents were less likely to support more spending on the NHS when activated to think about noncontributors, but particularly “unmotivated” noncontributors. Effects were modest, however, and more research should be done to confirm the findings before building on them. The difference between support for greater spending on the NHS when respondents were activated to think of “motivated” versus “unmotivated” noncontributors was marginally significant by standard thresholds. Moreover, support remained high across the board, reflecting that general public pressures on politicians to spend more may not change markedly. The likelihood of supporting “more” or “much more” spending on the NHS was 86.2 percent when noncontributions were not mentioned, 83.2 percent for “motivated” noncontributors, and 80.2 percent for “unmotivated” noncontributors.

In a set of empirical extensions, estimates revealed that presenting users with more severe medical conditions raised baseline support for spending, presumably by anchoring respondents to think about the NHS delivering more life-essential treatments. However, mentioning need did not alter our core results. Additionally, we found that conservatives (who may be more likely to emphasize a “self-reliance” narrative) were especially averse to more spending when activated to think of “unmotivated” noncontributors. We did not find that higher-income earners (who might have a greater personal stake in the tax base) or older respondents (who might be more likely to use the NHS) expressed significantly different preferences than other respondents. However, limited statistical power, combined with conditioning on non-experimentally-manipulated covariates, makes us cautious about interpreting these results.

Our study contributes to an extensive literature on public opinion toward government spending (Brooks and Manza 2007; Garritzmann, Häusermann, and Palier 2022) by suggesting that voters distinguish between the motivations of noncontributors when assessing UPGs. When goods that most voters themselves consume are at stake, they still support less spending when activated to think of “unmotivated” noncontributors. This outcome suggests that, while standard utility-maximizing models may powerfully predict democratic agency (Downs 1957), there is a critical social dimension (Tajfel 1974; Sigmund 2007) to how people support UPGs. Where concerns about upholding tacit social bargains exist, our finding sheds light on how voters tend to support universal versus particularist social expenditures (Korpi and Palme 1998; Rothstein 2001; Brady and Bostic 2015; van Oorschot et al. 2017).

In leveraging the NHS as an emblematic UPG to make inferences about other state provisions, our results also speak to a burgeoning literature on the politics of healthcare (Wendt, Mischke, and Pfeifer 2011; Missinne, Meuleman, and Bracke 2013; Carpenter 2015; Habibov et al. 2018; Busemeyer 2021). Our analysis most closely aligns with research examining how citizens support health provisions for individuals who fall victim to sickness versus joblessness (Jensen and Petersen 2017). A key finding is that citizens tend to view healthcare as a form of insurance against “as-if” randomly exposed risk, making it less of a wedge issue than unemployment benefits. We add to this research by showing that the motivations of users of the healthcare system—as measured by whether users are perceived to uphold their side of an implied social contract—further shape public opinion toward state health spending.

## The Universal Public Goods Trade-Off

Claims about the “deservingness” of government beneficiaries are salient in public policy, often leveraged by politicians, activists, and the media to argue either for or against more public spending (Zucchino 1999; Katz 2013; Romano 2018). From “strivers” to “skivers” (Williams 2013), motivational tropes can serve as forceful images for demands to either expand or retrench welfare. Study after study, for example, confirms that voters support fewer transfers (e.g., unemployment benefits, tax credits for the poor, food stamps) when noncontributors to the public tax base are viewed as lacking motivation to care for themselves (Gilens 1999; van Oorschot 2000; Appelbaum 2001; DeSante 2013). Concerns about motivations are often so strong that they can overwhelm both self-interest and other deeply held values informing support for means-tested entitlements (Petersen et al. 2011; Petersen 2012).

Yet even if voters support less welfare for “unmotivated” beneficiaries, it is unclear whether a similar dynamic applies to universal public goods (UPGs). Such goods—like universal healthcare—are distinctive in that they may have a redistributive component, but they are most characterized by being free and available to all citizens. This means that UPGs yield a particular tension. Lower spending on UPGs can ensure that “unmotivated” noncontributors receive fewer benefits. However, it can also erode the goods that most voters themselves use (or may use in the future). Consequently, voters face a trade-off. One option is to prioritize their preferred levels of UPGs by maintaining consistent support for these provisions, disregarding the motivations of noncontributors. Another option is to reduce their support for UPGs and undercut their ideal levels of these goods when they know that “unmotivated” citizens will consume them.

Seminal research (Olson 1965; Hardin 1971) indicates that individuals may act contrary to their own interests by penalizing “undeserving” beneficiaries who do not contribute to the public good. Certain game-theoretic models, for instance, show that the desire to avoid being a “sucker” can induce players to penalize noncooperators even when it clashes with selfish motivations (Osborne 2009). Laboratory experiments similarly find that social punishment can override self-interest when individuals perceive that others receive benefits without contributing proportionally (Ostrom, Walker, and Gardner 1992; Fehr and Gachter 2000, 2002). This research mostly concerns whether a beneficiary contributes to the public good. However, the noncontributor could be “motivated” or “unmotivated” depending on their reasons for not contributing, a key dimension in practical evaluations of UPGs, but one rarely made explicit.

For UPGs, this distinction matters because it is not obvious whether voters support less spending when noncontributors access goods primarily due to their noncontributions or because they make assumptions about motivations. On the one hand, voters might endure lower than their ideal levels of UPGs when considering noncontributors because their primary concern is whether recipients pay into the tax base. On the other hand, voters might endure lower than their ideal levels of UPGs because, in the absence of more information, they assume that some fraction are “unmotivated.” If voters could better gauge motivations, they may make sharper distinctions. Voters might support even less spending on UPGs when noncontributors are “unmotivated.” Especially because goods they themselves consume are at stake, they may also make more allowances if noncontributors are “motivated.”

Based on this distinction, we conceptualize two discrete ways in which voters can express spending preferences for UPGs when faced with “undeserving” users. *Contributions-only-based preferences* are concerned only with contributions to the underlying tax base. If other citizens do not contribute to the public good through financing UPGs, voters may support less than their preferred levels of these provisions. Voters are agnostic, however, about whether they perceive noncontributors to be “motivated” or “unmotivated.” Alternatively, *motivation-based preferences* distinguish between noncontributors. Voters care not only about a lack of contributions, but also their motivations. When noncontributors are viewed as “unmotivated,” voters should support lower than their preferred levels of UPGs, whereas they should be less inclined to do so when noncontributors are viewed as “motivated.”

## The Experiment

To test support for *contributions-only* or *motivation-based preferences*, we asked respondents in the UK to express their opinions about spending on an important UPG—the country’s universal National Health Service (NHS)—after being provided with different prompts describing fictional users. By randomly varying whether beneficiaries are presented as contributing to the tax system—and, if not, whether noncontributors are presented as “motivated” or “unmotivated”—we isolate the impact of these presentations in activating more or less support for spending on healthcare. Data were collected online from a panel of UK citizens 18 years of age and older in February 2018 (pre-COVID-19) by the survey firm Survey Sampling International (SSI, now Dynata). The sample was nationally representative by age, gender, and statistical region.^2^ A total of 3,000 respondents finished the survey, from a base of 3,505 eligible individuals who started the survey. The survey was not preregistered, and as such, may be interpreted as a plausibility probe.

## Test Case

**UK Case:** We examine the UK case because the concept of “deservingness” plays a salient role in public debates over state programming in the country. Numerous analyses suggest that distinctions between “motivated” and “unmotivated” beneficiaries have featured prominently in the nation’s public discourse and policy choices (BBC 2010; Behr 2012; *The Guardian* 2012; Lakasing 2015). Survey data corroborate that considerable percentages of the British public express concern about the level of state benefits afforded to “unmotivated” recipients. For example, in recent years, approximately 30 to 40 percent of Britons have agreed that “most people on the dole [unemployment benefits] are fiddling in one way or another.” Additionally, upwards of 50 percent of British citizens have expressed concern that government benefits for the unemployed are “too high and discourage work” (NatCen Social Research 2015).

**NHS:** We concentrate on the NHS because, despite having a redistributive component, it is regarded a paradigmatic UPG whose services are used widely across the population. The NHS is a universal, single-payer healthcare scheme where all citizens are automatically enrolled into the system and can use its services for free. In England alone, the NHS serves more than 300 million primary care patient visits each year (NHS 2020). The UK does have a small private healthcare insurance and provider system, but the overwhelming number of UK residents—across class and income brackets—rely on the NHS. This means that the NHS should be characterized by the UPG trade-off. Lower spending on the NHS would reduce the amount of resources devoted to “unmotivated” noncontributors, but reduce the ideal availability and quality of healthcare that most voters could personally access.

The NHS is also instructive because it comprises a large section of overall government spending in the UK—equating to nearly 10 percent of national income (The King’s Fund 2019b). This not only makes healthcare a high-stakes policy area for the UK government, but it also should increase its salience among the electorate and generate informed and stable opinions over its financing. Amid recent concerns over hospital overcrowding (Yeginsu 2018) and other financial strains on the UK health sector due to aging and rising costs (Triggle 2018), the NHS has been a particularly high-profile issue in public debates, with politicians on both the left and the right making it a central part of their campaign platforms. In the 2019 UK general election, both Conservative and Labour leaders called for increased funding for the NHS in their party manifestos, highlighting its centrality to national politics.^3^

Finally, the NHS’s affirmed popularity makes it a “hard test” for shifting public opinion. Polling data, for example, show that 77 percent of Britons think “the NHS is crucial to British society” and that the UK “must do everything we can to maintain it” (Wellings 2017). Unlike in some countries, such as the United States, where debates over government regulation of healthcare are polarized (Smith 2015), the NHS receives broad cross-partisan support. In recent years, the NHS has been likened to everything from a “national treasure” (West 2013) to a “national religion” (Field 2016) to the country’s “greatest national asset” (Stubley 2020) to “a veritable Golden Calf” (Herron 2020). To the extent that activating voters to think about “unmotivated” noncontributors lowers relative support for the NHS even amid this status, such impacts should be at least as large when applied to other UPGs where public support is more fragile.

## Noncontributions and “Deservingness”

Our experiment manipulates whether NHS users are presented as noncontributors to the tax base—and if so, whether they are “motivated” or “unmotivated.” We designed the vignettes to resemble the sorts of characterizations of NHS users to which voters might be exposed from politicians, political actors, and the media (Zucchino 1999; Katz 2013; Romano 2018). Responses can be interpreted as the effect of activating citizens to think of individual users of the NHS as noncontributors based on their motivations.

**Noncontributions:** To confirm that voters are generally averse to subsidizing “noncontributors,” we first randomly vary mentioning (or not) noncontributions of NHS users based on their employment status. In the UK, approximately 99 percent of NHS funding comes from taxes (The King’s Fund 2019a)^4^: 81 percent from general taxation, and another 18 percent from National Insurance (NI) contributions (which can be understood as a tax on the employed). Of the amount that comes from general taxation, about 48 percent comes from taxes tied to employment status (Miller and Roantree 2017).^5^ Overall, this means that about 57 percent of all NHS funding comes from sources linked to employment. Therefore, although other forms of taxation partially fund the NHS—and even those without jobs pay incidental contributions to the NHS—unemployment serves as a reasonable marker of a noncontributor.

**Motivations:** For our main tests, we vary the motivations of NHS users presented as noncontributors. Although there are many ways to define and measure motivation (Lerner, Miller, and Holmes 1976; Feather 2002; Watkins-Hayes and Kovalsky 2016; Meuleman, Roosma, and Abts 2020), our construct is based on an implied social contract requiring that citizens contribute proportionally to society based on what they can reasonably pay. When citizens violate that tacit agreement—by not working and paying taxes when they could—this entitlement is likely to be seen as a breach of social norms (Fong, Bowles, and Gintis 2006; Petersen 2015). In short, we define motivation in terms of whether NHS users work or willfully exploit the contributions of others, thereby violating tenets of reciprocity. For simplicity, we dichotomize motivations, such that users are presented as either “motivated” or “unmotivated.”

## Main Treatments

Respondents were randomly assigned to one of three main groups based on whether NHS users contributed to the tax base—and if not, whether they were “motivated” or “unmotivated.” All the vignettes (see Supplementary Material C for full text) opened by explaining that the NHS faces major challenges, such as hospital overcrowding, and described heavy usage of the NHS. The vignettes then introduced two fictional users of the NHS—Patrick Smith and George Peterson—who were recently treated for medical conditions. In *Treatment 1* (“motivated” noncontributor), the text presents Smith and Peterson—as representative of many other NHS users—as unemployed and includes quotes from them expressing eagerness to find work and to pay taxes that fund the NHS.

The U.K.’s National Health Service (NHS) faces big challenges, including significant overcrowding at hospitals. Many unemployed people in the U.K., who do not pay income taxes, use the NHS.

One such person is Patrick Smith, 37, from Birmingham, who says, “I’m actively applying for jobs and hope soon to pay my fair share to support the NHS.” Another is George Peterson, 47, from London, who says, “I’m trying my best to find work and to contribute meaningfully to the NHS.”

In *Treatment 2* (“unmotivated” noncontributor), the text again presents Smith and Peterson—as representative of many other NHS users—as unemployed but includes quotes from them expressing a lack of interest in finding work and feeling entitled to NHS services.

The U.K.’s National Health Service (NHS) faces big challenges, including significant overcrowding at hospitals. Many unemployed people in the U.K., who do not pay income taxes, use the NHS.

One such person is Patrick Smith, 37, from Birmingham, who says, “I’m not too concerned about finding a new job, since the NHS will always support me.” Another is George Peterson, 47, from London, who says, “I’m entitled to NHS services anyway, so I’m not in a rush to find work.”

In the *Control* condition, the text does not mention the employment status of NHS users, does not highlight Smith and Peterson as unemployed, and does not include any quotes.

The U.K.’s National Health Service (NHS) faces big challenges, including significant overcrowding at hospitals. Many people in the U.K. use the NHS.

One such person is Patrick Smith, 37, from Birmingham. Another is George Peterson, 47, from London.

Because our treatments do not include normative judgments about the motivations of the noncontributors, the language is likely mild compared to what voters might receive from activists with express agendas over cutting or expanding UPGs. Deliberate efforts to play up the motivation (or lack thereof) of noncontributors—through explicitly using these terms or by invoking other phrases and concepts designed to activate more positive or negative views about the beneficiaries (Shildrick 2018)—might sway public opinion more. There are numerous instances of the UK media reporting on such language, including with regard to healthcare.^6^ To the extent this is the case, the treatments should only underestimate how much the motivation of users affects voter support for spending on UPGs.

## Subtreatments: Need of Users

In line with standard studies of government spending, we also condition on the needs of beneficiaries (Reeskens and van Oorschot 2013; Meuleman, Roosma, and Abts 2020). Because voters should support higher spending to the extent that provisions are considered essential to users or there is deprivation requiring reliance on government services, we expect greater needs of users to boost levels of support for healthcare spending. In particular, baseline support for NHS spending may increase with the presentation of more severe medical needs, due to the anchoring effect (Tversky and Kahneman 1974; Mussweiler and Strack 2000) of respondents considering the NHS delivering more life-essential treatments. To the extent that respondents react similarly in relative terms to “motivated” and “unmotivated” noncontributors regardless of need, it affirms the robustness of our findings.

We presented NHS users as suffering from medical needs ranging from benign (common cold) to serious (hip problems) to emergency (heart failure).^7^ At the end of the vignettes, we randomly added information about the needs of the users. In *Subtreatment A* (“benign”), the text notes that Smith and Peterson—as representative of other NHS users—were treated for benign medical conditions.^8^

Last month, both Patrick and George—along with many other [unemployed] people—were treated by NHS doctors for common colds that would have left them feeling temporarily uncomfortable if left untreated.

In *Subtreatment B* (“serious”), the text refers to serious medical conditions.

Last month, both Patrick and George—along with many other [unemployed] people—were treated by NHS doctors for hip problems that would have made them unable to walk if left untreated.

In *Subtreatment C* (“emergency”), the text refers to emergency medical conditions.

Last month, both Patrick and George—along with many other [unemployed] people—were treated by NHS doctors for heart failure that would have killed them on the spot if left untreated.

## Summary of Vignettes

A summary of the vignettes, totaling nine different conditions, is provided in Table 1.

**Table 1.** Summary of vignettes.

Treatment 1A	Motivated noncontributor	Benign
Treatment 1B		Serious
Treatment 1C		Emergency
Treatment 2A	Unmotivated noncontributor	Benign
Treatment 2B		Serious
Treatment 2C		Emergency
Control A	Control	Benign
Control B		Serious
Control C		Emergency

## Nonexperimental Moderators: Respondent Ideology and Material Self-Interest

In addition to our experimental treatments, we also extend our analysis by conditioning on standard respondent covariates proxying: 1) voter ideology; and 2) material self-interest.

**Ideology.** Ideology-based models of spending preferences suggest that political persuasion features prominently in the willingness of voters to support state expenditures (Feldman and Zaller 1992; Alesina and Glaeser 2004; Lelkes and Sniderman 2014). Ideology eschews a standard rational-choice logic in formulating attitudes toward government spending in favor of opinions based more on personal values and conceptions of fairness. We predict that conservative or right-leaning voters, who emphasize and embrace a “self-reliance” narrative (Feather 1984; Lakoff 2016), should be more concerned both with the contributions of beneficiaries and their motivations. Individuals who tilt to the right politically are more likely to resent or to judge harshly others who express feelings of entitlement, making them especially likely to react negatively when presented with “unmotivated” noncontributors who use UPGs.

**Material Self-Interest.** Conventional redistribution models assume that material incentives to support state spending are moderated by how much voters pay into the tax base (Meltzer and Richard 1981; Iversen and Soskice 2001) and how much they benefit (Busemeyer, Goerres, and Weschle 2009). The more that voters see themselves as having a stake in the tax base that funds a UPG, the less they should want resources spent on noncontributors, and particularly “unmotivated” noncontributors. We predict that higher-income earners, who pay proportionally higher taxes and have more “skin in the game,” might be more averse to subsidizing “unmotivated” noncontributors given greater personal resources at stake. Conversely, older populations, because of their greater likelihood of requiring medical care, might resist reducing spending on healthcare, regardless of the motivations of other users.

## Dependent Variable: Attitudes toward Spending

The dependent variable is support for government spending on the NHS, which we measure with the question: “Would you like to see more or less government spending than there is today on the NHS?” *Contributions-only* or *motivation-based preferences* entail supporting lower spending than voters would otherwise prefer. To ensure that respondents express a general preference for government spending on the NHS, the question does not ask specifically about spending to help the users in the vignettes. For simplicity in interpreting our main results, we dichotomize the DV and estimate unweighted linear probability models, coding “Government should spend much more” or “Government should spend more” as 1 and 0 otherwise.^9^ As robustness checks, we keep the full variation in our DV and estimate ordered logit models (and, alternatively, OLS models), all unweighted, and we also look at the dependent variable of supporting “less” or “much less” spending. As described below, we do not find any substantive differences between our main results and alternative models.

## Results

### Contribution of Users

We first check whether respondents activated to think of noncontributors as users support less spending on the NHS, irrespective of the motivations of those noncontributors.^10^ Model 1 of table 2 reports these results. As predicted, the coefficient on *Non−contributor* is negative and statistically significant (*p* = .001), suggesting that respondents support less spending on the NHS when activated to think of noncontributors as users.^11^ This finding is consistent with individuals being more reluctant to fund UPGs when considering beneficiaries who do not pay into the tax system. As shown in Supplementary Material figure A1, high levels of support exist across the board, reflecting the status of healthcare as a valence issue. The portion of respondents who prefer “more” or “much more” spending on the NHS is 81.7 percent for the noncontributor condition, compared to 86.2 percent for the control group.

**Table 2.** Support for increased spending on the NHS, by user contribution and “motivation.”

	(1)	(2)	(3)	(4)
	LPM	OLogit	LPM	Ologit
Noncontributor	−0.0450 (0.001)	−0.176 (0.014)		
Unmotivated			−0.0296	−0.125
noncontributor			(0.087)	(0.142)
Control			0.0302	0.114
			(0.061)	(0.169)
Constant	0.862 (0.000)		0.832 (0.000)	

*Note:* Table shows unstandardized coefficients from linear probability models (Models 1 and 3) and ordered logit models (Models 2 and 4) using robust standard errors and unweighted data with no individual covariates (*p*-values shown in parentheses). In Models 1 and 2, the omitted category is the control condition, and all noncontributors are lumped together (we do not disaggregate by “motivation”). In Models 3 and 4, the omitted category is the “motivated noncontributor” condition.

## Motivation of Users

For our main tests, we disaggregate the effect of the noncontributor treatment by motivation.^12^ In Model 3 of table 2, we find a negative and marginally significant coefficient on *UnmotivatedNon−contributor* (*p* = .09), meaning that respondents activated to consider noncontributors as “unmotivated” rather than “motivated” support less spending on the NHS.^13^ These findings align with the *motivation-based preferences hypothesis*.^14^ As revealed in Supplementary Material figure A2, the proportion of respondents who favor “more” or “much more” spending on the NHS is 80.2 percent for the “unmotivated” noncontributor condition, 83.2 percent for the “motivated” noncontributor condition, and 86.2 percent for the control. In comparing responses to the “unmotivated” and “motivated” noncontributor treatments, the difference in support for increased spending of 3.0 percentage points is modest, yet still notable in the context of a UPG with large baseline support, where the question is about overall (not targeted) spending, and where the treatments offer no explicit normative judgments of the beneficiaries.^15^ Also notable is the variation of a full six percentage points between respondents receiving the “unmotivated” noncontributor treatment and the control.^16^

## Need of Users

Next, we test whether support for more spending on the NHS is conditioned by the perceived health needs of users. Table 3 reports these results. In Model 1, we do not find statistically significant coefficients on either *Noncontributor X Serious* (*p* = .42) or *Noncontributor X Emergency* (*p* = .90), suggesting that the severity of the medical conditions with which users are presented does not alter our core findings. However, respondents directionally support greater baseline spending when presented with users suffering from more severe medical conditions (main effects of “serious” and “emergency” are positive). This is consistent with our expectation of an anchoring effect whereby citizens are activated to think of the NHS providing more expensive, life-essential services.^17^ We do flag here the limitations to statistical power in analyzing the conditional effects by need. The standard errors of the estimates for interaction terms in table 3 are about 50 percent larger than for the main effects in table 2, as each of the subgroups contain fewer respondents. As such, we interpret these models as more suggestive.^18^

**Table 3.** Support for increased spending on the NHS, by user contribution, “motivation,” and need.

	(1)	(2)	(3)	(4)
	LPM	OLogit	LPM	OLogit
Noncontributor	−0.0530	−0.0687		
	(0.040)	(0.579)		
Serious	0.0261	0.196	0.0635	0.299
	(0.348)	(0.157)	(0.026)	(0.038)
Emergency	0.0539	0.536	0.0175	0.214
	(0.043)	(0.000)	(0.563)	(0.152)
Noncontributor *×* Serious	0.0284	0.00226		
	(0.418)	(0.990)		
Noncontributor *×* Emergency	−0.00434	−0.344		
	(0.899)	(0.053)		
Unmotivated noncontributor			−0.0450	−0.0452
			(0.159)	(0.769)
Unmotivated noncontributor *×* Serious			−0.0178	-0.203
			(0.677)	(0.337)
Unmotivated noncontributor *×* Emergency			0.0641	−0.0421
			(0.138)	(0.843)
Control			0.0305	0.0465
			(0.305)	(0.744)
Control *×* Serious			−0.0373	−0.103
			(0.349)	(0.607)
Control *×* Emergency			0.0364	0.322
			(0.366)	(0.119)
Constant	0.835 (0.000)		0.805 (0.000)	
Observations	3000	3000	3000	3000

*Note:* Table shows unstandardized coefficients from linear probability models (Models 1 and 3) and ordered logit models (Models 2 and 4) using robust standard errors and unweighted data with no individual covariates (*p*-values shown in parentheses). In Models 1 and 2, the omitted category is the control-benign condition, whereas in Models 3 and 4, the omitted category is the “motivated noncontributor-benign” condition.

## Respondent Ideology and Material Self-Interest

Finally, we analyze whether our results are moderated by the ideology and material self-interests of voters. Results are presented in table 4. In Model 1, we find a negative and marginally significant coefficient on the interaction *Unmotivated Noncontributor X Conservative* (*p* = .09), indicating that conservatives do support comparatively less spending on the NHS when presented with “unmotivated” as opposed to “motivated” noncontributors. By contrast, we find no statistically significant coefficients on the interactions *Unmotivated Noncontributor X Income* (Model 2) or *Unmotivated Noncontributor X > *65 (Model 3), suggesting that income and being older (over age 65) do not affect responses to “unmotivated” noncontributors.^19^ These results imply that concerns related to the “self-reliance” narrative—more than paying a larger amount of taxes or being especially dependent on healthcare services—appear to underlie our main findings. As shown in Supplementary Material figure A5, the number of conservatives who support “more” or “much more” spending is 76.1 percent when receiving the “unmotivated” noncontributor treatment, compared to 83.7 percent for the “motivated” noncontributor treatment (a spread of 7.6 percentage points). For liberals, those numbers are 89.7 and 91.4 percent, respectively (a difference of only 1.7 percentage points). As before, however, we are cautious about interpreting these effects because of constraints on statistical power. The standard errors of the estimates for interaction terms in table 4 are between the same size (for *Conservative*) to 50 percent larger (*Income* and *Age*) than for the main effects in table 2.^20^

**Table 4.** Support (dichotomous) for increased spending on the NHS, by user contribution, “motivation,” ideology, age, and income subgroups.

	(1)	(2)	(3)
Unmotivated noncontributor	−0.000962	−0.0373	−0.0254
	(0.967)	(0.138)	(0.178)
Control	0.0241	0.0307	0.0302
	(0.0160)	(0.0161)	(0.0161)
Unmotivated noncontributor *×* Conservative	−0.0476		
	(0.095)		
Unmotivated noncontributor *×* Income		0.000278	
		(0.655)	
Unmotivated noncontributor *×>*65 years			−0.0184
			(0.610)
Conservative	−0.0880		
	(0.000)		
Income		−0.00119	
		(0.000355)	
*>*65 years			−0.00306
			(0.873)
Constant	0.898	0.869	0.831
	(0.000)	(0.000)	(0.000)
Observations	3000	3000	3000

*Note:* Table shows unstandardized coefficients from linear probability models using robust standard errors and unweighted data with no individual covariates (*p*-values shown in parentheses). Conservatives are coded as those who self-identify as being “slightly to the right,” “to the right,” or “extremely to the right.” We also add a dichotomous variable for moderates, making liberals the reference group. Income is in thousands of pounds. The questions used are in the Supplementary Material.

## Conclusion

We fielded a nationally representative survey experiment in the UK to test whether voters support less spending on universal public goods (UPGs) when noncontributors to the tax base are perceived as “unmotivated.” Analyzing the NHS, the country’s universal single-payer healthcare system, we found that respondents favor less spending on UPGs when activated to think of noncontributors as users. Support is lowest, however, when noncontributors are presented as “unmotivated” and respondents making the evaluations are conservatives apt to prioritize the importance of self-reliance. Although the effects are modest, these findings hold when conditioning on user needs, as proxied by the severity of medical conditions. Overall, results indicate that when goods that most voters consume are on the line, they discern between “motivated” and “unmotivated” noncontributors in supporting spending on UPGs.

Our study adds to an extensive public opinion literature on “deservingness” and government spending by examining how voters approach the UPG trade-off. Unlike with means-tested entitlements, UPGs generate a specific tension. Lower spending on UPGs decreases benefits for “unmotivated” noncontributors, but at the cost of reducing the ideal level of goods that most voters demand. Prior research shows that individuals will sometimes reduce their preferred spending levels when faced with “undeserving” users. Yet studies largely overlook whether preferences are conditioned not just by whether users fail to contribute to the tax base, but whether they are “motivated” or “unmotivated.” We clarify that voters depart more from their preferred spending levels on UPGs when activated to think of “unmotivated” noncontributors who are perceived as not upholding their side of a tacit social contract.

Our nonpreregistered predictions make it important to re-establish and confirm these results. Future research should examine whether similar patterns of support hold for the NHS in the post-COVID-19 era^21^ and for UPGs across other policy areas, such as education, where the profile of beneficiaries may differ.^22^ Scholars could also test whether results replicate outside the UK, especially in settings where spending on public services tends to be more or less generous and connected to individual contributions (e.g., the Nordic countries versus the United States), where average perceptions of the motivation of the unemployed differ, and where perceived motivation may interact with other social divides (e.g., race, gender, age). Our results suggest that efforts to portray “undeserving” users as “motivated” or “unmotivated” may not only influence voter support for means-tested entitlements, but also for UPGs that are free and open to all citizens.

## Supplementary Material

nfad007_Supplementary_Data

## Data Availability

Underlying data and documentation are available in the Harvard Dataverse at https://doi.org/10.7910/DVN/ASSTWS.

## Footnotes

1 Although there are some differences in how the term “undeserving” is invoked in scholarship and popular discourse, we use the term broadly to refer to noncontributors to the tax base. By this definition, the “undeserving” may be either “motivated” or “unmotivated,” depending on their willingness to exert effort.

2
Supplementary Material table B1 shows demographic means for respondents in our sample relative to national UK means for the covariates we include in subsequent regressions. The only substantial deviation from national averages is that respondents are considerably more likely not to be employed. Values of the covariates are balanced across treatment groups (one out of sixteen differences in means is statistically significant), indicating successful randomization.

3 See https://vote.conservatives.com/our-plan and https://labour.org.uk/manifesto-2019/rebuild-our-public-services/.

4 About 1 percent of the NHS is funded through patient charges.

5 These taxes include those on income and wealth, including property taxes. Of the remainder, 37 percent comes from VAT or other consumption taxes, 10 percent comes from corporate taxes, and 5 percent comes from other taxes, including devolved taxes and environmental levies.

6 In the UK, efforts by the media and politicians to present the poor as “motivated” (Wolff 2019) or “unmotivated” (Smith 2010) are common. With the NHS specifically, the notion of “abusers” of the system extends to numerous areas, as evidenced by articles such as “The NHS and Demos—It’s a Bad Idea to Stigmatise the Poor” (*The Independent*) (Board 2013), “Foreign Patients Cheat NHS out of £30MILLION in One Year” (*The Daily Express*) (Hall 2016), or “Put Feckless Patients at the Back of NHS Queue” (*The Daily Telegraph*) (Foges 2018).

7 In contrast to many policy areas, where need could be considered subjective, what constitutes minor or major medical conditions should be less ambiguous for the healthcare case.

8 As denoted by the brackets, when respondents received *Treatment 1* or *2*, we re-emphasized that the users were unemployed, whereas we made no mention of this in the *Control* condition.

9 The other options were: “Government should spend the same as now,” “Government should spend less,” and “Government should spend much less.”

10 We collapse Treatments 1 and 2 and estimate linear probability models: *Yi = α + β1Noncontributori + εi*, where *Noncontributor* denotes noncontributors, regardless of their “motivation” and the severity of their medical conditions (the *Control* is the omitted term).

11 These results hold in Model 2 of table 2 (using ordered logit regression); in Models 1 and 2 of Supplementary Material table B2 (where we include individual-level covariates); and in Model 1 of Supplementary Material table B3 (where we estimate OLS models using the full 1–5 variation in the dependent variable). When we use support for “less” or “much less” spending as the dependent variable in Models 1 and 2 of Supplementary Material table B4, we find directionally similar coefficients, although they are less precisely estimated because of how few voters support actually reducing expenditures.

12 We estimate the following linear probability model: Yi=α+β1Controli+β2UnmotivatedNoncontributori+εi, where *UnmotivatedNoncontributor* represents the “unmotivated” noncontributor treatment, again irrespective of the severity of medical conditions, and *Control* represents the randomization arm where no employment information was provided (the “motivated” noncontributor treatment is the omitted category).

13 Results are again comparable when using ordered logit regression (Model 4 of table 2); when including individual-level covariates (Models 3 and 4 of Supplementary Material table B2); and when estimating OLS models with the complete 1–5 range in the dependent variable (Model 2 of Supplementary Material table B3). As before, when changing the dependent variable to support for “less” or “much less” spending, we find directionally similar (though less precisely estimated) coefficients (Models 3 and 4 of Supplementary Material table B4).

14 The *p-*value of the main effect suggests there is only a 9 percent probability that we would incorrectly reject the null hypothesis of there being no actual effect of the “unmotivated” noncontributor treatment in activating respondents to reduce preferences for more spending.

15 The difference in support between those receiving the “unmotivated” and “motivated” noncontributor treatments is about the same size as the difference in support between those who receive the “motivated” noncontributor treatment and the control group, or about three percentage points. See Model 3 of table 2.

16 In Supplementary Material table B5, we also analyze support for other DVs that might be expected to mirror reduced levels of support for more spending—namely, efforts to ration NHS services through co-payments, caps on individual usage, and prioritizing users who pay income taxes. See Supplementary Material C for the full set of questions. In Models 1, 3, and 5, we find that the noncontributor treatment prompts respondents to be more supportive of rationing (although these effects are only statistically significant for caps). In Models 2, 4 and 6, we find that this is particularly true when respondents are activated to think about noncontributors as “unmotivated” (where the effects are statistically significant for all the outcomes).

17
Supplementary Material figure A3 summarizes the likelihood of respondents supporting “more” or “much more” spending for the full nine treatment-control combinations.

18 Results are similar in Models 2 and 4 of table 3 (where we estimate ordered logit models) and in Models 3 and 4 of Supplementary Material table B3 (where we estimate OLS models using the full 1–5 scale in the dependent variable). Supplementary Material table B6 and corresponding Supplementary Material figure A4 show results using as the dependent variable support for “less” or “much less” spending. The effects line up almost symmetrically with those of support for more spending.

19 The only other main regression we estimated (but do not report) included an interaction with a dichotomous employment variable. The results were not statistically significant and capture a similar effect as income.

20 We also acknowledge the limitations of conditioning on non-experimentally-manipulated variables.

21 Research suggests that online experiments conducted during the COVID-19 pandemic yield similar results to those conducted prior to the outbreak (Peyton, Huber, and Coppock 2021).

22 See, for example, Ansell (2010); Busemeyer, Garritzmann, and Neimanns(2020).

## References

[nfad007-B1] Alesina Alberto , Glaeser Edward L. 2004. Fighting Poverty in the US and Europe: A World of Difference. New York: Oxford University Press.

[nfad007-B2] Ansell Ben W. 2010. From the Ballot to the Blackboard: The Redistributive Political Economy of Education. Cambridge: Cambridge University Press.

[nfad007-B3] Appelbaum Lauren D. 2001. “The Influence of Perceived Deservingness on Policy Decisions Regarding Aid to the Poor.” Political Psychology 22:419–42.

[nfad007-B4] BBC. 2010. “The Deserving or Undeserving Poor?” *BBC*, November 18. https://www.bbc.com/news/magazine-11778284.

[nfad007-B5] Behr Rafael. 2012. “Shirkers vs. Strivers.” *New Statesman*, November 29.

[nfad007-B6] Bienkov Adam. 2019. “Boris Johnson Said Patients Should Have to Pay to Use the NHS So They Will ‘Value’ It More.” *Business Insider*, December 7. https://www.businessinsider.com/boris-johnson-said-patients-charged-to-use-the-nhs-2019-12.

[nfad007-B7] Brady David , Bostic Amie. 2015. “Paradoxes of Social Policy: Welfare Transfers, Relative Poverty, and Redistribution Preferences.” American Sociological Review 80:268–98.

[nfad007-B8] Brooks Clem , Manza Jeff. 2007. Why Welfare States Persist: The Importance of Public Opinion in Democracies. Chicago: University of Chicago Press.

[nfad007-B9] Busemeyer Marius , Garritzmann Julian L., Neimanns Erik. 2020. A Loud but Noisy Signal? Public Opinion and Education Reform in Western Europe. Cambridge: Cambridge University Press.

[nfad007-B10] Busemeyer Marius R. 2021. “Financing the Welfare State in Times of Extreme Crisis: Public Support for Health Care Spending During the Covid-19 Pandemic in Germany.” Journal of European Public Policy 30:21–40.

[nfad007-B11] Busemeyer Marius R. , Goerres Achim, Weschle Simon. 2009. “Attitudes towards Redistributive Spending in an Era of Demographic Ageing: The Rival Pressures from Age and Income in 14 OECD Countries.” Journal of European Social Policy 19:195–212.

[nfad007-B12] Carpenter Daniel. 2015. “Is Health Politics Different?” Annual Review of Political Science 15:287–311.

[nfad007-B13] Cavaillé Charlotte , Trump Kris-Stella. 2015. “The Two Facets of Social Policy Preferences.” Journal of Politics 77:146–60.

[nfad007-B14] DeSante Christopher D. 2013. “Working Twice as Hard to Get Half as Far: Race, Work Ethic, and America’s Deserving Poor.” American Journal of Political Science 57:342–56.

[nfad007-B15] Downs Anthony. 1957. “An Economic Theory of Political Action in a Democracy.” Journal of Political Economy 65:135–50.

[nfad007-B17] Feather Norman T. 1984. “Protestant Ethic, Conservatism, and Values.” Journal of Personality and Social Psychology 46:1132–41.

[nfad007-B16] Feather Norman T. 2002. Value, Achievement and Justice: Studies in the Psychology of Deservingness. New York: Kluwer. 10.1207/s15327957pspr0302_1 15647142

[nfad007-B18] Fehr Ernst , Gachter Simon. 2000. “Cooperation and Punishment in Public Goods Experiments.” American Economic Review 4:980–94.

[nfad007-B19] Fehr Ernst , Gachter Simon 2002. “Altruistic Punishment in Humans.” Nature 415:137–40. 11805825 10.1038/415137a

[nfad007-B20] Feldman Stanley , Zaller John. 1992. “The Political Culture of Ambivalence: Ideological Responses to the Welfare State.” American Journal of Political Science 36:268–307.

[nfad007-B21] Field Frank. 2016. “The NHS Is Our National Religion—But There’s No Miracle Funding Cure.” *The Guardian*, February 23. https://www.theguardian.com/society/2016/feb/23/nhs-religion-funding-mutual.

[nfad007-B22] Fong Christiana M. , Bowles Samuel, Gintis Herbert. 2006. “Strong Reciprocity and the Welfare State.” In Handbook on the Economics of Giving, Reciprocity and Altruism, vol. 2, edited by Kolm Serge-Christophe Ythier Jean Mercier, 1439–64. Amsterdam: Elsevier.

[nfad007-B23] Foges Clare. 2018. “Put Feckless Patients at the Back of NHS Queue.” *The Times*, April 2. https://www.thetimes.co.uk/article/put-feckless-patients-at-the-back-of-nhs-queue-5hnlqqstg.

[nfad007-B24] Garritzmann Julian L. , Häusermann Silja, Palier Bruno, eds. 2022. The World Politics of Social Investment (Volume I): Welfare States in the Knowledge Economy. Oxford: Oxford University Press.

[nfad007-B25] Gilens Martin. 1999. Why Americans Hate Welfare: Race, Media, and the Politics of Antipoverty Policy. Chicago: University of Chicago Press.

[nfad007-B26] Glaze Ben. 2019. “Boris Johnson Called for NHS Patients to Pay to See GP or Call an Ambulance.” *The Mirror* December 3. https://www.mirror.co.uk/news/politics/boris-johnson-called-nhs-patients-21018692.

[nfad007-B27] The Guardian. 2012. “Rich and Poor: Deserving and Undeserving.” *The Guardian*, January 27. https://www.theguardian.com/commentisfree/2012/jan/27/rich-poor-deserving-undeserving.

[nfad007-B28] Habibov Nazim , Auchynnikava Alena, Luo Rong, Fan Lida. 2018. “Who Wants to Pay More Taxes to Improve Public Health Care?” International Journal of Health Planning and Management 33:e944–e959. 29992623 10.1002/hpm.2572

[nfad007-B29] Hall Macer. 2016. “Foreign Patients Cheat NHS out of £30MILLION in One Year.” *The Express* December 20. https://www.express.co.uk/news/uk/745490/foreign-patients-cheat-nhs-30-millions-one-year.

[nfad007-B30] Hardin Russell. 1971. “Collective Action as an Agreeable n-Prisoners’ Dilemma.” Behavioral Science 16:472–81.

[nfad007-B31] Herron Gareth. 2020. “The NHS Has Taken over the Church as the UK’s National Religion.” *News Letter*, April 18. https://www.newsletter.co.uk/news/opinion/letters/nhs-has-taken-over-church-uks-national-religion-2542512.

[nfad007-B32] The Independent Editorial Board. 2013. “Editorial: The NHS and Demos—It’s a Bad Idea to Stigmatise the Poor.” *The Independent*, March 11. https://www.independent.co.uk/voices/editorials/editorial-the-nhs-and-demos-it-s-a-bad-idea-to-stigmatise-the-poor-8529821.html.

[nfad007-B33] Iversen Torben , Soskice David. 2001. “An Asset Theory of Social Policy Preferences.” American Political Science Review 95:875–93.

[nfad007-B34] Jensen Carsten , Petersen Michael Bang. 2017. “The Deservingness Heuristic and the Politics of Health Care.” American Journal of Political Science 61:68–83.

[nfad007-B35] Johnson Boris. 1995. “Why Shouldn’t We Pay a Fiver for Accidentally Summoning an Ambulance. It’s Politics, Stupid.” *The Spectator*, September 23. http://archive.spectator.co.uk/article/23rd-september-1995/6/politics.

[nfad007-B36] Johnson Boris. 2019. “Remarks at: ‘The Queen’s Speech and Associated Background Briefing, on the Occasion of the Opening of Parliament on Monday 14 October 2019.’” https://assets.publishing.service.gov.uk/government/uploads/system/uploads/attachment_data/file/839370/Queen_s_Speech_Lobby_Pack_2019_.pdf.

[nfad007-B37] Katz Michael B. 2013. The Undeserving Poor: America’s Enduring Confrontation with Poverty. 2nd ed. Oxford: Oxford University Press.

[nfad007-B38] The King’s Fund. 2019a. “How the NHS Is Funded.” September 26. https://www.kingsfund.org.uk/projects/nhs-in-a-nutshell/how-nhs-funded.

[nfad007-B39] The King’s Fund. 2019b. “Key Facts and Figures about the NHS.” November 18. https://www.kingsfund.org.uk/audio-video/key-facts-figures-nhs.

[nfad007-B40] Korpi Walter , Palme Joakim. 1998. “The Paradox of Redistribution and Strategies of Equality: Welfare State Institutions, Inequality, and Poverty in the Western Countries.” American Sociological Review 63:661–87.

[nfad007-B41] Lakasing Edin. 2015. “The Concept of the ‘Undeserving Poor’: Pejorative Stereotypes and Worsening Inequalities Undermine Welfare Reform.” British Journal of General Practice: The Journal of the Royal College of General Practitioners 65:652. 26622021 10.3399/bjgp15X687925 PMC4655721

[nfad007-B42] Lakoff George. 2016. Moral Politics: How Liberals and Conservatives Think. 3rd ed. Chicago: University of Chicago Press.

[nfad007-B43] Lelkes Yphtach , Sniderman Paul M. 2014. “The Ideological Asymmetry of the American Party System.” British Journal of Political Science 46:825–44.

[nfad007-B44] Lerner Melvin J. , Miller Dale T., Holmes John G. 1976. “Deserving and the Emergence of Forms of Justice.” Advances in Experimental Social Psychology 9:113–62.

[nfad007-B45] Lupu Noam , Pontusson Jonas. 2011. “The Structure of Inequality and the Politics of Redistribution.” American Political Science Review 105:1–21.

[nfad007-B46] Luttmer Erzo F. P. 2001. “Group Loyalty and the Taste for Redistribution.” Journal of Political Economy 109:500–528.

[nfad007-B47] Meltzer Allen H. , Richard Scott F. 1981. “A Rational Theory of the Size of Government.” Journal of Political Economy 89:914–27.

[nfad007-B48] Meuleman Bart , Roosma Femke, Abts Koen. 2020. “Welfare Deservingness Opinions from Heuristic to Measurable Concept: The CARIN Deservingness Principles Scale.” Social Science Research 85. [Online] 10.1016/j.ssresearch.2019.102352 31789191

[nfad007-B49] Miller Hellen , Roantree Barra. 2017. “Tax Revenues: Where Does the Money Come from and What Are the Next Government’s Challenges?” IFS Briefing Note 198, Figure 2. https://ifs.org.uk/sites/default/files/output_url_files/BN198.pdf.

[nfad007-B50] Missinne Sarah , Meuleman Bart, Bracke Piet. 2013. “The Popular Legitimacy of European Healthcare Systems: A Multilevel Analysis of 24 Countries.” Journal of European Public Policy 23:231–47.

[nfad007-B51] Mussweiler Thomas , Strack Fritz. 2000. “The Use of Category and Exemplar Knowledge in the Solution of Anchoring Tasks.” Journal of Personality and Social Psychology 78:1038–52. 10870907 10.1037//0022-3514.78.6.1038

[nfad007-B52] NatCen Social Research. 2015. “British Social Attitudes 32: Benefits and Welfare.” https://www.bsa.natcen.ac.uk/media/38977/bsa32_welfare.pdf.

[nfad007-B89] NHS 2020. “NHS Five Year Forward View: Primary Care.” https://www.england.nhs.uk/five-year-forward-view/next-steps-on-the-nhs-fiveyear-forward-view/primary-care/.

[nfad007-B53] OECD. 2019. “Health at a Glance: OECD Indicators.” 10.1787/4dd50c09-en.

[nfad007-B54] Olson Mancur. 1965. The Logic of Collective Action. Cambridge, MA: Harvard University Press.

[nfad007-B55] Osborne Martin J. 2009. An Introduction to Game Theory. Oxford: Oxford University Press.

[nfad007-B56] Ostrom Elinor , Walker James, Gardner Roy. 1992. “Covenants with and without a Sword: Self-Governance Is Possible.” American Political Science Review 86:404–17.

[nfad007-B57] Petersen Michael Bang. 2012. “Social Welfare as Small-Scale Help: Evolutionary Psychology and the Deservingness Heuristic.” American Journal of Political Science 56:1–16. 22375300 10.1111/j.1540-5907.2011.00545.x

[nfad007-B58] Petersen Michael Bang. 2015. “Evolutionary Political Psychology: On the Origins and Structure of Heuristics and Biases in Politics.” Political Psychology 36:45–76.

[nfad007-B59] Petersen Michael Bang , Slothuus Rune, Stubager Rune, Togeby Lise. 2011. “Deservingness versus Values in Public Opinion on Welfare: The Automaticity of the Deservingness Heuristic.” European Journal of Political Research 50:25–52.

[nfad007-B60] Peyton Kyle , Huber Gregory A., Coppock Alexander. 2021. “The Generalizability of Online Experiments Conducted During the COVID-19 Pandemic.” Journal of Experimental Political Science.

[nfad007-B61] Rand David G. , Ohtsuki Hisashi, Nowak Martin A. 2009. “Direct Reciprocity with Costly Punishment: Generous Tit-for-Tat Prevails.” Journal of Theoretical Biology 256:45–57. 18938180 10.1016/j.jtbi.2008.09.015 PMC2614626

[nfad007-B62] Reeskens Tim , van Oorschot Wim. 2013. “Equity, Equality, or Need? A Study of Popular Preferences for Welfare Redistribution Principles across 24 European Countries.” Journal of European Public Policy 20:1174–95.

[nfad007-B63] Roemer John E. , Lee Woojin, van der Straeten Karine. 2007. Racism, Xenophobia, and Distribution: Multi-Issue Politics in Advanced Democracies. New York: Russell Sage Foundation.

[nfad007-B64] Romano Serena. 2018. Moralising Poverty: The “Undeserving” Poor in the Public Gaze. London: Routledge.

[nfad007-B65] Rothstein Bo. 2001. “The Universal Welfare State as a Social Dilemma.” Rationality and Society 13:213–33.

[nfad007-B66] Shayo Moses. 2009. “A Model of Social Identity with an Application to Political Economy: Nation, Class, and Redistribution.” American Political Science Review 103:147–74.

[nfad007-B67] Shildrick Tracy. 2018. Poverty Propaganda: Exploring the Myths. Bristol, UK: Policy Press.

[nfad007-B68] Sigmund Karl. 2007. “Punish or Perish? Retaliation and Collaboration among Humans.” Trends in Ecology & Evolution 22:593–600. 17963994 10.1016/j.tree.2007.06.012

[nfad007-B69] Smirnov Oleg. 2007. “Altruistic Punishment in Politics and Life Sciences: Climbing the Same Mountain in Theory and Practice.” Perspectives on Politics 5:489–501.

[nfad007-B70] Smith Iain Duncan. 2010. “Speech: ‘Universal Credit: Welfare That Works.’” https://www.gov.uk/government/speeches/universal-credit-welfare-that-works.

[nfad007-B71] Smith Samantha. 2015. “From the Very Start, Sharp Partisan Divisions over Obamacare.” Pew Research Center, January 25. https://www.pewresearch.org/fact-tank/2015/06/25/from-the-very-start-sharp-partisan-divisions-over-obamacare/.

[nfad007-B72] Stubley Peter. 2020. “Coronavirus: Boris Johnson Praises NHS as Country’s Greatest National Asset after Saying ‘He Could Have Gone Either Way.’” *The Independent*, April 13. https://www.independent.co.uk/news/uk/politics/coronavirus-boris-johnson-health-news-hospital-nhs-video-tweet-a9461616.html.

[nfad007-B73] Tajfel Henri. 1974. Intergroup Behavior, Social Comparison and Social Change. Ann Arbor: Katz-Newcomb Lectures.

[nfad007-B74] Topple Steve. 2020. “Johnson’s Latest Act of Mind-blowing Hypocrisy Nearly Broke Twitter.” *The Canary*, May 2. https://www.thecanary.co/trending/2020/05/02/johnsons-latest-act-of-mind-blowing-hypocrisy-nearly-broke-twitter/.

[nfad007-B75] Triggle Nick. 2018. “10 Charts That Show Why the NHS Is in Trouble.” BBC, May 24. https://www.bbc.com/news/health-42572110.

[nfad007-B76] Tversky Amos , Kahneman Daniel. 1974. “Judgment under Uncertainty: Heuristics and Biases.” Science (New York, N.Y.) 185:1124–31. 17835457 10.1126/science.185.4157.1124

[nfad007-B77] van Oorschot Wim. 2000. “Who Should Get What, and Why? On Deservingness Criteria and the Conditionality of Solidarity among the Public.” Policy and Politics 28:33–48.

[nfad007-B78] van Oorschot Wim , Meuleman Bart. 2014. “Popular Deservingness of the Unemployed in the Context of Welfare State Policies, Economic Conditions and Cultural Climate.” In How Welfare States Shape the Democratic Public: Policy Feedback, Participation, Voting, and Attitudes, edited by Kumlin Staffan, Stadelmann-Steffen Isabelle. Cheltenham, UK: Edward Elgar.

[nfad007-B79] van Oorschot Wim , Roosma Femke, Meuleman Barte, Reeskens Tim, eds. 2017. The Social Legitimacy of Targeted Welfare: Attitudes towards Welfare Deservingness. Cheltenham, UK: Edward Elgar.

[nfad007-B80] Watkins-Hayes Celeste , Kovalsky Elyse. 2016. “The Discourse of Deservingness: Morality and the Dilemmas of Poverty Relief in Debate and Practice.” In The Oxford Handbook of the Social Science of Poverty, edited by Brady David, Burton Linda, 193–220. Oxford: Oxford University Press.

[nfad007-B81] Wellings Dan. 2017. “What Does the Public Think about the NHS?” *The King’s Fund*, September 16. https://www.kingsfund.org.uk/publications/what-does-public-think-about-nhs.

[nfad007-B82] Wendt Claus , Mischke Monika, Pfeifer Michaela. 2011. Welfare States and Public Opinion: Perceptions of Healthcare Systems, Family Policy and Benefits for the Unemployed and Poor in Europe. Cheltenham, UK: Edward Elgar.

[nfad007-B83] West Michael. 2013. “The NHS Is a National Treasure. We Must Continue to Guard It.” *The Guardian*, September 11. https://www.theguardian.com/healthcare-network/2013/sep/11/nhs-study-culture-behaviour.

[nfad007-B84] Williams Zoe. 2013. “Skivers v. Strivers: The Argument that Pollutes People’s Minds.” *The Guardian*, January 9. https://www.theguardian.com/politics/2013/jan/09/skivers-v-strivers-argument-pollutes.

[nfad007-B85] Wolff Jonathan. 2019. “The Myth of the Undeserving Poor.” *The New Statesman*, November 22. https://www.newstatesman.com/politics/2019/11/the-myth-of-the-undeserving-poor.

[nfad007-B86] Yamagishi Toshio. 1986. “The Provision of a Sanctioning System as a Public Good.” Journal of Personality and Social Psychology 51:110–16.

[nfad007-B87] Yeginsu Ceylan. 2018. “N.H.S. Overwhelmed in Britain, Leaving Patients to Wait.” *New York Times*, January 3. https://www.nytimes.com/2018/01/03/world/europe/uk-national-health-service.html.

[nfad007-B88] Zucchino David. 1999. Myth of the Welfare Queen. New York: Simon & Schuster.

