# Supplementary material for: “Deservingness” and Public Support for Universal Public Goods: A Survey Experiment
Source: Public Opin Q. 2023 Apr 3;87(1):44–68. doi: 10.1093/poq/nfad007 (PMC10824553; doi:10.1093/poq/nfad007)
Supplement: nfad007_Supplementary_Data [file nfad007_supplementary_data.pdf]

Supplementary Material: "Deservingness" of Beneficiaries and Support for Universal Public Services: A Survey Experiment on the UK's National Health Service

Thomas Gift (UCL) and Carlos X. Lastra-Anadón (IE)

October 13, 2022

Appendix A: Additional Figures and Tables

Figure A1: Likelihood of supporting "more" or "much more" spending on the NHS, by user contribution, "deservingness" and need

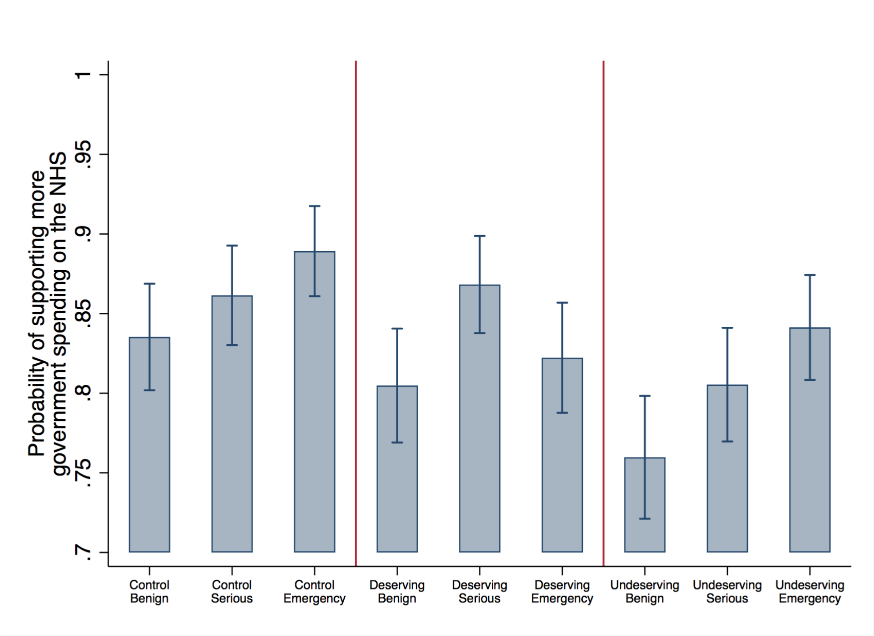

Table A1: Comparison with U.K. national figures and balance of the covariates across the different treatment groups

	(0) U.K. Mean	(1) Full sample Mean	(2) Control Mean	(3) Motivated NC Mean	(4) (3) - (2)	(5) Unmotivated NC Mean	(6) (5) - (2)
Female	0.508	0.512	0.511	0.519	0.008	0.506	-0.005
		(0.500)	(0.500)	(0.500)	[0.737]	(0.500)	[0.840]
Age	48.332	46.376	45.822	47.059	1.237	46.248	0.426
		(17.602)	(17.594)	(17.447)	[0.115]	(17.759)	[0.590]
Parent	N.A.	0.617	0.604	0.631	0.027	0.616	0.012
		(0.486)	(0.489)	(0.483)	[0.221]	(0.487)	[0.570]
White	0.870	0.907	0.905	0.907	0.002	0.910	0.005
		(0.290)	(0.293)	(0.291)	[0.884]	(0.286)	[0.694]
Born in U.K.	0.86	0.927	0.924	0.935	0.011	0.921	-0.003
		(0.261)	(0.265)	(0.247)	[0.340]	(0.270)	[0.807]
University graduate	0.384	0.363	0.374	0.325	-0.049*	0.389	0.015
		(0.481)	(0.484)	(0.469)	[0.022]	(0.488)	[0.501]
Not employed	0.244	0.437	0.438	0.436	-0.002	0.436	-0.002
		(0.496)	(0.496)	(0.496)	[0.944]	(0.496)	[0.913]
Household income	28,400	32,215	32,125	31,647	-478	32,872	747
		(25,262)	(24,587)	(25,177)	[667]	(26.012)	[509]
Observations		3,000	1,000	999	1,999	1,001	2,001

NC= non-contributor. Displays mean values of covariates in each of the treatment groups and their difference with the control. Household income is in thousands of pounds. Source for gender, age, ethnicity and place of birth is ONS (2018a), employment and income is ONS (2018b), university graduate is ONS (2020a), and immigration share in the local authority is ONS (2020b). In parentheses, standard deviations for means and, in square brackets, p values for differences in means. $^{+}$ $p < 0.10$, * $p < 0.05$, ** $p < 0.01$, *** $p < 0.001$

Table A2: Support for increased spending on the NHS, using ordered logit models

	(1)	(2)
Non-Contributor	-0.176**	
	(0.0717)	
Undeserving		-0.125
		(0.0853)
Control		0.114
		(0.0830)
Observations	3000	3000

Coefficients from ordered logit models with no individual covariates. Robust standard errors in parentheses. * $p < 0.10$, ** $p < 0.05$, *** $p < 0.01$

Table A3: Support for increased spending on the NHS, by user contribution and "motivation", including individual covariates

	(1) LPM	(2) OLogit	(3) LPM	(4) OLogit
Non-contributor	-0.0452***	-0.179**		
	(0.001)	(0.013)		
Unmotivated			-0.0592	-0.130
Non-contributor			(0.104)	(0.128)
Control			0.0524	0.114
			(0.134)	(0.174)
Female	0.0397***	0.161**	0.0748**	0.160**
	(0.005)	(0.027)	(0.015)	(0.027)
Age	-0.000169	-0.00564**	-0.00188*	-0.00575**
	(0.720)	(0.020)	(0.064)	(0.018)
Children	0.0200	0.171**	0.0677**	0.171**
	(0.180)	(0.024)	(0.035)	(0.024)
White	0.0792***	0.284**	0.148**	0.287**
	(0.005)	(0.039)	(0.012)	(0.037)
UK Native	-0.0243	-0.0533	-0.0522	-0.0581
	(0.368)	(0.693)	(0.343)	(0.668)
Some college	0.0206	0.139*	0.0592*	0.145**
	(0.150)	(0.054)	(0.053)	(0.046)
Unemployed	0.00239	0.00195	0.0136	0.00420
	(0.882)	(0.981)	(0.695)	(0.960)
Income (tsd)	-0.00107***	-0.00950***	-0.00369***	-0.00949***
	(0.001)	(0.000)	(0.000)	(0.000)
Observations	3000	3000	3000	3000

Coefficients from linear probability models (Models 1 and 3) and ordered logit models (Models 2 and 4) with no individual covariates. In Models 1 and 2, the omitted category is the control condition, and all non-contributors are lumped together (we do not disaggregate by "motivation"). In Models 3 and 4, the omitted category is the "motivated non-contributor" condition. p values in parentheses. * $p < 0.10$, ** $p < 0.05$, *** $p < 0.01$

Table A4: Support (dichotomous) for increased spending on the NHS, by user contribution, "deservingness", and partisanship and income subgroups

	(1)	(2)
Non-contributor Undeserving	-0.0451**	-0.0606**
	(0.0215)	(0.0274)
Control	0.0304	-0.0174
	(0.0201)	(0.0259)
Non-contributor undeserving X Left	0.0348	
	(0.0333)	
Non-contributor undeserving X Income		0.00102
		(0.000732)
Control X Left	-0.0228	
	(0.0314)	
Control X Income		0.00151**
		(0.000703)
Left	0.162***	
	(0.0276)	
Income		-0.00193***
		(0.000525)
Constant	0.745***	0.893***
	(0.0205)	(0.0185)
Observations	3000	3000

Coefficients from linear probability models with no individual covariates, except Model 1 includes a "moderate" covariate. Income is in thousands of dollars. Robust standard errors in parentheses. * $p < 0.10$, ** $p < 0.05$, *** $p < 0.01$

Table A5: Support for rationing NHS services

	(1) Co-payments	(2) Co-payments	(3) Caps	(4) Caps	(5) Waitlists	(6) Waitlists
Non-contributor	0.0130		0.0495***		0.00400	
	(0.497)		(0.006)		(0.819)	
Control		0.0256		-0.0263		0.0187
		(0.244)		(0.201)		(0.348)
Unmotivated non-contributor		0.0771***		0.0463**		0.0454**
		(0.000)		(0.029)		(0.025)
Constant	0.421***	0.395***	0.291***	0.317***	0.283***	0.264***
	(0.000)	(0.000)	(0.000)	(0.000)	(0.000)	(0.000)
Observations	3000	3000	3000	3000	3000	3000

Support for all rationing types dichotomized, with option indicating no support coded as 0, and those indicating conditional or unconditional support coded as 1. Coefficients from linear probability models with no individual covariates. In Models 1, 3, and 5, the omitted category is the control condition, whereas in Models 2, 4, and 6, the omitted category is the "motivated non-contributor" condition. p values in parentheses. * $p < 0.10$, ** $p < 0.05$, *** $p < 0.01$

Appendix B: Survey Instrument

Vignettes

The survey instrument included the following vignettes:

General Treatment Text
The U.K.'s National Health Service (NHS) faces big challenges, including significant overcrowding at hospitals. Many unemployed people in the U.K., who do not pay income taxes, use the NHS.

One such person is Patrick Smith, 37, from Birmingham, who says, "[Deserving/Undeserving (Quote 1)]." Another is George Peterson, 47, from London, who says, "[Deserving/Undeserving (Quote 2)]."

Last month, both Patrick and George - along with many other unemployed people - were treated by NHS doctors for [Benign/Serious/Emergency].

General Control Text
The U.K.'s National Health Service (NHS) faces big challenges, including significant overcrowding at hospitals. Many people in the U.K. use the NHS.

One such person is Patrick Smith, 37, from Birmingham. Another is George Peterson, 47, from London.

Last month, both Patrick and George - along with many other people - were treated by NHS doctors for [Benign/Serious/Emergency].

<u>Primes</u>
Deserving (Quote 1): I'm actively applying for jobs and hope soon to pay my fair share to support the NHS
Deserving (Quote 2): I'm trying my best to find work and to contribute meaningfully to the NHS
Undeserving (Quote 1): I'm not too concerned about finding a new job, since the NHS will always support me
Undeserving (Quote 2): I'm entitled to NHS services anyway, so I'm not in a rush to find work
Benign: common colds that would have left them feeling temporarily uncomfortable if left untreated
Serious: hip problems that would have made them unable to walk if left untreated
Emergency: heart failure that would have killed them on the spot if left untreated

Dependent variable questions

Spending
Would you like to see more or less government spending than there is today on the NHS?

Government should spend much more
Government should spend more
Government should spend the same as now
Government should spend less
Government should spend much less

Co-Payments
Should users of the NHS be required to pay a portion of the cost of treatment out of pocket?

Yes, everyone should pay a portion of the cost of treatment out of pocket
Yes, but only those who do not pay income taxes should pay a portion of the cost of treatment out of pocket
No, no one should pay a portion of the cost of treatment out of pocket

Caps
Should there be a cap on the number of times someone can use the NHS in a given year without being charged?

Yes, everyone should have a cap on the number of times they can use the NHS in a given year without being charged
Yes, but only those who do not pay income taxes should have a cap on the number of times they can use the NHS in a given year without being charged
No, everyone should be able to use the NHS as many times as they request without being charged

Waitlists
When it comes to receiving healthcare through the NHS, should the government prioritize citizens who pay income taxes over those who do not pay (for example, moving those who do not pay income taxes to the back of waiting lists)?

Yes
No

Non-experimental moderator questions

Conservative (Right, including moderates)
Below is a 7-point scale on which the political views that people might hold are arranged from extremely to the left to extremely to the right. Where would you place yourself on this scale?

1 Extremely to the left
2 To the left
3 Slightly to the left

4 Moderate, middle of the road
5 Slightly to the right
6 To the right
7 Extremely to the right

Income
What is your total gross annual household income from all sources?

Under £10,000
£10,000 to £19,999
£20,000 to £29,999
£30,000 to £39,999
£40,000 to £49,999
£50,000 to £59,999
£60,000 to £69,999
£70,000 to £79,999
£80,000 to £89,999
£90,000 to £99,999
Over £100,000
Would rather not say

>65 years
What is your age?

Under 18
18-24
25-34
35-44
45-54
55-64
65+
